# Altered structural and causal connectivity in frontal lobe epilepsy

**DOI:** 10.1186/s12883-019-1300-z

**Published:** 2019-04-25

**Authors:** Benjamin Klugah-Brown, Cheng Luo, Rui Peng, Hui He, Jianfu Li, Li Dong, Dezhong Yao

**Affiliations:** The Clinical Hospital of Chengdu Brain Science Institute, MOE Key Lab for Neuroinformation, Center for Information in Medicine, High-Field Magnetic Resonance Brain Imaging Key Laboratory of Sichuan Province, School of Life Science and Technology, University of Electronic Science and Technology of China, No. 4, Section 2, North Jianshe Road, Chengdu, People’s Republic of China

**Keywords:** Frontal lobe epilepsy, Effective connectivity, Causality, Functional magnetic resonance imaging, Gray matter volume

## Abstract

**Background:**

Albeit the few resting-state fMRI neuroimaging studies in frontal lobe epilepsy (FLE) patients, these studies focused on functional connectivity. The aim of this current study was to examine the effective connectivity based on voxel-based morphometry in FLE patients.

**Methods:**

Resting-state structural and functional magnetic resonance imaging (fMRI) data were acquired from 19 FLE patients and 19 age and gender-matched healthy controls using the 3.0 Tesla magnetic resonance imaging (3.0 T MRI). The investigations were done by acquiring the structural information through voxel-based morphometry, then based on the seed obtained, Granger causality analysis was used to evaluate the causal flow of the designated seed to and from other significant voxels.

**Results:**

Our results showed altered structural and effective connectivity. Compared with healthy controls, FLE patients showed reduced grey matter volume in bilateral putamen and right caudate as well as altered causality with increased, and decreased causal outflow from the right caudate (seed region) to inferior frontal gyrus-triangular, from bilateral putamen (seed regions) to right middle frontal gyrus and frontal gyrus medial-orbital representing the frontal executive areas, respectively. Also, significantly increased and decreased inflow from left calcarine to right caudate and from cerebellum_6 and vermis_6 to bilateral putamen, respectively. Moreover, we found that the causal alterations to and from the seed regions (from vermis_6 to right putamen and from left putamen to right middle frontal gyrus) negatively correlated with clinical scores (duration of epilepsy).

**Conclusions:**

The findings point to the impairment within the executive and motor-controlled system including the cerebellum, frontal, caudate and putamen regions in FLE patients. These results would therefore enhance our understanding of structural and effective mechanisms in FLE.

## Background

Epilepsy is a frequent type of neurological disorder with frontal lobe epilepsy (FLE) as the second most prevalent focal epilepsies following temporal lobe epilepsy (TLE). FLE It is known to commonly occur briefly during sleep or wake, and often originates from the frontal lobes [1]. FLE is known to occur in about 20 to 30% of focal epilepsy sufferers [2], however, FLE neuroimaging studies compared with TLE is less investigated.

Though methods in neuroimaging have greatly improved, FLE may be misdiagnosed as one of the following: psychiatric disorder, non-epileptic seizures or sleep disorder [3], partly owing to the complex nature of the frontal lobe and the difficulty in detecting seizure onset. Nonetheless, seizures leading to FLE may fall within the following locations: supplementary motor area [4], primary motor cortex [1], medial cingulate gyrus, orbitofrontal, or frontopolar regions [5], Dorsolateral cortex and Operculum. These areas are involved in motor modulation and sensory functions, including distribution and signal inputs from other part of motor related regions. Seizure onset of FLE is usually accompanied by deficits in sensorimotor across the fronto-parietal lobe through the Basal ganglia (BG). As an important brain area, BG has been known to modulate sensorimotor and motor activities in humans. It mainly consists of the striatum, the subthalamic nucleus and the substantia nigra with parallel, separated and linked-loop projections between it and functionally distinct regions of the cerebral cortex [6, 7]. In epilepsy, structures in the BG has been widely investigated in many of functional [8] and structural [9] studies, signifying its’ crucial role for the modulation of epileptic activity. In addition, altered functional connectivity involving the frontal lobe through the BG to the cerebellum in children with FLE have also been reported [10], suggesting the crucial role these regions play during seizures in FLE.

Recently, voxel-based morphometry (VBM) has been used to define the subtle structural alteration of the brain on the scale of voxel-by-voxel or seed-to-voxel in different neuropsychiatric disorders. Structural analyses in epilepsy have shown changes in the grey matter volume (GMV) of cortical and subcortical nuclei, the volume changes is in regards to decreasing or increasing grey matter, as well as the spreading of epileptic activity to areas away from the seizure zones [11, 12]. Furthermore, structural analyses in epilepsy have revealed changes in the grey matter volume of epilepsy patients [13], these studies continue to show decreasing and/or increasing cortical GMV changes. It is worth nothing that, relatively few of these researches typically focused on FLE, such as in [14, 15].

Connectivity methods measure the statistical relationship between or among unique timeseries of anatomical areas of the brain [16], this allows researchers to investigate patterns of connections as well as their inter-dependencies including the causal flow within the circuitry of the brain. Statistically, researchers can explore whether one timeseries can be relied upon to predict another through Granger Causality Analysis (GCA). GCA is often estimated with multivariate auto-regression, which is based on effective connectivity (EC) concepts [17]. Therefore, our choice of using GCA as a method to analyze connectivity of the VBM was motivated by the fact that GCA can provide the dynamic and causality of structural fMRI signals in the brain of subjects under study [18]. Thus, enhancing our understanding about the statistical influence of an observed area on other regions without prior knowledge. In addition, we have applied this method in children with benign childhood epilepsy [19].

Moreover, several researches have been conducted using GCA to investigate the information flow, Hamilton and colleagues [20] used multivariate approach of GCA to show the spatial relation in the abnormal resting-state networks of major depressive disorder. In 2010 Liao et al. [21] applied GCA to study the effective connectivity pattern of the amygdala in patients with social anxiety disorders. Also, as a method of choice, GCA can be used to understand these predictabilities of one area to the other of neurological disease such as epilepsy. It is worth nothing that, epilepsy is profoundly complex. Besides, in both EEG and MRI modalities the aberrant connectivity that underlies seizure mechanisms have been studied using effective connectivity (EC). In recent times, the study of specific kinds of epilepsy are being investigated, Ji et al. [22] employed GCA to investigate EC in patients with mesial temporal lobe epilepsy. Another study by Wei et al. [23] found significant differences between IGE patients and healthy controls within connectivity graphs, which is consistent with the time and frequency domain of GCA. Thus, GCA would be a valuable method in investigating neurological disorders as well as provide a decent understanding of their mechanisms. Here, we investigated the structural alterations and also evaluated the causality of statistically significant areas of GMV between FLE patients and healthy controls using GCA.

## Material and method

### Subjects

In this study, we used resting-state fMRI experimental data, including a total of 19 FLE patients (9 females; mean age = 24.2 years; standard deviation = 9.5 years; age range = 13–51 years; number of patients with unilateral interictal epileptic discharges (IED) = 4 (left) and 6(right), number of patients with bilateral IED = 9) recruited from the Clinical Hospital of Chengdu Brain Science Institute (CBSI), University of Electronic Science and Technology of China (UESTC). All patients were diagnosed by neurologists based on the clinical information in accordance with the International League Against Epilepsy (ILAE) guidelines (Engel & International League Against Epilepsy (ILAE), 2001 and also based on clinical history, 24-h video-EEG recording, ictal semiology and routine imaging (CT and/or MRI). No anatomical abnormalities in FLE patients were found using routine examinations of CT and MRI scanning. All patients under-went 24-h (overnight including the sleep period) scalp video-EEG recordings (EEG–9100 K, Nihon Kohden, Tokyo, Japan). For EEG,16 electrodes were distributed according to 10–20 international standard system, and the sampling rate was set at 256 Hz. Using24-h EEG recording, 3/19 FLE patients showed burst of sharpwaves; 10/19 patients showed sparse sharp waves; 4/19 patients showed sparse sharp-slow waves; and 2/19 patients showed burst of sharp-slow waves. And, 10/19 patients demonstrated interictal discharges in the frontal regions; 6/19 patients demonstrated interictal discharges in the frontotemporal areas; and 3/19 patients demonstrated discharges in the front-central regions. All patients received antiepileptic drug (AED) treatments with regular outpatient follow-up Detailed demographic information (such as; age of epilepsy onset, Interictal EEG, seizure type, medication, family history of epilepsy) can be found in Table 1. The dataset used in this study is same as our previous research [24]. In addition, 19 age and gender-matched, healthy participants were also recruited (5 females; mean age = 23.9 years; standard deviation = 8.9 years; age range = 11–41 years). All approaches and the study procedure were approved by the local Ethics Committee of UESTC. We also required that written approval forms be submitted by each participant. All subjects provided written consent to participate in this study. Part of the consent included the exact information about the scanning procedure and psychological assessment. The study was approved by the Ethics Committee of the clinical hospital of Chengdu Brain Science Institute (CBSI) in accordance with the Declaration of Helsinki.

**Table 1:** Demographic and clinical information of FLE patients

No.	Age of onset	Duration of epilepsy	Interictal EEG	Seizure type	Medication	Family history of epilepsy
1.	16	8	Right; Burst of theta sharp waves; FP2, F4, F8	SGTCS	CBZ/PIR	Brother
2.	12	10	Bilateral; Sparse sharp-slow waves; FP1, FP2, F3, F4, F8	SPS	TPM	Sister
3.	38	3	Left; Sparse sharp waves; F7, T3	SPS	TPM/OXC/TCM	–
4.	16	7	Bilateral; Burst of sharp-slow waves; FP2, C3, F3, F4, F8,	CPS	LTG	–
5.	11	4	Bilateral; Burst of sharp waves; C3, F7, F4, F8	SPS	*	Brother
6.	10	16	Right; Sparse sharp waves; F4, F8	CPS	VPM/PIR	–
7.	3	11	Bilateral; Sparse sharp waves; FP2, F7, T3	CPS	CBZ/GAS	Sister
8.	7	11	Bilateral; Sparse sharp waves; FP2, F7, F8, T3	SPS	TPM/OXC	–
9.	18	14	Bilateral; Sparse sharp waves; FP1, F7, T3, F8	CPS	VPA	–
10.	10	4	Right; Sparse sharp and sharp-slow waves; FP2, F4, C4	SPS	OXC/PIR	Brother
11.	13	10	Bilateral; Sparse sharp waves; FP2, F4, F7, F3	SGTCS	VPM/PIR	–
12.	7	11	Right; Sparse sharp waves; F4, F8	CPS	LEV	–
13.	8	17	Right; Burst of sharp waves; F4, F8, T4	SPS	CBZ	–
14.	12	9	Left; Sparse sharp-slow waves; FP1, F3	SPS	CBZ/TCM	–
15.	18	10	Bilateral; Burst of sharp-slow waves; FP1, FP2, F3, F4, F8	SGTCS	VPM/OXC	–
16.	11	3	Left; Sparse sharp-slow waves; FP1, F3	CPS	OXC/TCM	–
17.	3	28	Right; Sparse sharp waves; FP2, F4	CPS	VPA/TPM	Sister
18.	51	0.5	Left; Sparse sharp waves; F3, F7, T3	SGTCS	**	–
19.	10	9	Bilateral; Sparse sharp waves; FP1, FP2, F4, F7	CPS	VPA	Sister

*VPA*: valproic acid; *VPM*: valpromide; *LTG*: lamotrigine; *CBZ*: carbamazepine; *TPM*: topiramate; *PIR*: piracetam; *OXC*: oxcarbazepine; *LEV*: levetiracetam; *GAS*: gastrodin; *TCM*: traditional Chinese medicine; *CPS*: complex partial seizure; *SPS*: simple partial seizure; *SGTCS*: secondary generalized tonic-clonic seizure; *: has no medication for about 2 months; **: drug-naive; M: male; F: female

### MRI acquisition

All MRI data were collected using an MRI scanner (3.0 T, Discovery MR750, GE, USA) in The Clinical Hospital of Chengdu Brain Science Institute of UESTC. T1-weighted anatomical images were collected using a three-dimensional fast spoiled gradient-echo (3D FSPGR) sequence, and the scanning parameters were as follows: slices = 152; TR/TE = 6.008 ms/1.984 ms; field of view = 256 × 256 mm2; flip angle = 9°; matrix size = 256 × 256 and slice thickness = 1 mm (no gap). The functional images were collected using a gradient-echo echo-planar imaging sequence. The scanning parameters were as follows: slices = 35; TR/TE = 2000 ms/30 ms; field of view = 240 × 240 mm2; flip angle = 90°; matrix size = 64 × 64 and thickness = 4 mm. A total of 255 volumes were obtained over a 510-s period. During the resting-state fMRI scanning, all subjects were explicitly instructed to close their eyes and relax without falling asleep.

### Structural data analysis

The acquired data were transformed from DICOM to Nifti format through the SPM8 toolbox in the MATLAB R2014. All resting-state imaging data were analyzed through standard steps of VBM analysis [25]. The Statistical Parametric Mapping (SPM, http://www.fil.ion.ucl.ac.uk/spm/) was used for this analysis. The following steps were employed; (1) we check all images for artifacts, and reoriented so that the image origins were set at the anterior commissure. T1-weighted images were then segmented into gray matter, white matter (WM) and cerebrospinal fluid (CSF), and total volume of gray matter which was used to estimate the true volume of the tissue was obtained within the space for each subject. (2) We then used DARTEL method to define the optimal registration of individual segments to a group mean template. The segments were based on the Jacobian determents for the correction of volume changes in nonlinear normalization. Subsequently, we normalized the segmented images to the Montreal Neurological Institute (MNI152) template. Next, the images were modulated to correct for volume changes caused by non-linear normalization. Finally, we smoothed the segmented gray matter after the modulation by Full width at half maximum (FWHM) 8 mm Gaussian kernel. (3) Here, the normalized volumes of gray matter, WM volume and whole brain were compared among the two groups, respectively. A gray matter optimal threshold mask, created from all subjects, was applied to eliminate voxels of nongray matter. (4) Statistical analysis was applied in SPM8 for comparing the gray matter volume between FLE patients and control by two sample t-test, age and gender were as covariates of no interest (*p* < 0.05, FWE corrected).

### Functional data preprocessing

For functional images, the first five (5) volumes were discarded to remove the T1 saturation effects, followed by slice timing, realignment, spatial normalization (3 × 3 × 3 mm^3^) and smoothing [8-mm full-width at half maximum (FWHM)] representing the preprocessing steps. Our analysis was performed by using a combination of toolboxes, including the fMRI toolbox found in the SPM8 software and (http://www.fil.ion.ucl.ac.uk/spm/software/spm8/), neuroscience information toolbox [26]. Data collected were thresholded at translation < 2 mm and rotation < 2° to avoid extreme head motion, which is known to introduce noise. The removal of the possible nuisance was done as follows: nuisance signals, which included linear trend, head motion, and the individual means of the white matter and cerebrospinal fluid signals, were excluded from the fMRI data through multiple linear regression analysis; Besides, framewise displacement (FD) was evaluated in the two groups [27]. We did not regress global signal as this was suspected to introduces anti-correlations and reduces the actual signal being analysed. The FD for each participant was evaluated using the following formula;

1 $$ FD=\frac{1}{T-1}\sum \limits_{i=2}^T\sqrt{{\left|\Delta  {d}_{x_i^1}\right|}^2+{\left|\Delta  {d}_{y_i^1}\right|}^2+{\left|\Delta  {d}_{z_i^1}\right|}^2+{\left|\Delta  {d}_{x_i^2}\right|}^2+{\left|\Delta  {d}_{y_i^2}\right|}^2+{\left|\Delta  {d}_{z_i^2}\right|}^2} $$

where T is the number of the fMRI time points, and ${x}_i^1/{x}_i^2,{y}_i^1/{y}_i^2$ and ${z}_i^1/{z}_i^2$ are translations/rotations at the *i*^*th*^ time point in the *x*, *y* and *z* directions, respectively; $\Delta  {d}_{x_i^1={x}_i^1-{x}_{i-1}^1}$.

### Granger causality analysis

Based on the following definitions; suppose there exist two indeterminate events *X*(*t*) and *Y*(*t*) and the subsequent values of *X*(*t*) is to be forecasted, using only the previous values of *X*(*t*) and the combination of the previous values *X*(*t*) and *Y*(*t*), our interest was to find out whether the acquired information of *Y*(*t*) can adequately predict *X*, such that *Y* can be said to have a causal influence on *X*, [28].

Mathematically;

Auto regression representation:

2 $$ {Y}_t={\sum}_{k=1}^p{b}_k{Y}_{\left(t-k\right)}+{\varepsilon}_t $$

3 $$ \mathit{\operatorname{var}}\left({\varepsilon}_t\right)={Q}_1 $$

4 $$ {X}_t={\sum}_{k=1}^pb{\prime}_k{X}_{\left(t-k\right)}+\varepsilon {\prime}_t $$

5 $$ \mathit{\operatorname{var}}\left({\varepsilon}_t^{\prime }={R}_1\right) $$

Where *ε*_*t*_ and ${\varepsilon}_t^{\prime }$ represent the residuals; *Q*_1_ and *R*_1_ represents the variances of the residuals respectively.

Joint regressive representation:

6 $$ {Y}_t={\sum}_{k=1}^p{A}_k{X}_{\left(t-k\right)}+{\sum}_{k=1}^p{B}_k{Y}_{\left(t-k\right)}+{\mu}_t $$

7 $$ \mathit{\operatorname{var}}\left({\mu}_t\right)={Q}_2 $$

8 $$ {X}_t={\sum}_{k=1}^p{A}_k^{\prime }{Y}_{\left(t-k\right)}+{\sum}_{k=1}^p{B}_k^{\prime }{X}_{\left(t-k\right)}+{\mu}_t^{\prime } $$

9 $$ \mathit{\operatorname{var}}\left({\mu}_t^{\prime}\right)={R}_2 $$

Where *μ*_*t*_ and *μ*′_*t*_ represents the residuals of the joint regressive formulation; *Q*_2_ and *R*_2_ represents the variances of the residuals respectively. *X*_*t*_ and *Y*_*t*_ represent the two events (time series) in time *t*. *X*_(*t* − *k*)_ and *Y*_(*t* − *k*)_ indicates the time series at the time *t* − *k*, *p* represents the number of lagged time point. The GCA will output *A*_*k*_, *A*′_*k*_ as the signed path coefficient maps and *B*_*k*_, *B*′_*k*_ as the autoregression coefficient maps.

Finally, to calculate the magnitude of causality to-and-from the two time-series;

10 $$ {F}_{x\to y}=\mathit{\ln}\frac{Q_1}{Q_2} $$

11 $$ {F}_{y\to x}=\mathit{\ln}\frac{R_1}{R_2} $$

Net-direction;

12 $$ \varDelta F=\left({F}_{y\to x}\right)-\left({F}_{x\to y}\right) $$

Descriptively, each of the parameter in Eq. 10 and Eq. 11 is derived by taking the log of a specific variance ratio between the obtained residuals of the regression of *X* plus the Y (past description) as well as the residuals of Y. Moreover, equations, *F*_*X* → *Y*_ or *F*_*X* ← *Y*_ shows the magnitude of causality of *X* or *Y* given the prediction of *Y* or *X*. Finally, the measure of flow of information calculated using the Δ*F* signifies the net influence of the direction of causality, if Δ*F* is positive then the net direction of causality is from *X*_*t*_ to *Y*_*t*_ and vice versa.

In this study, based on the structural significant difference within the GMV, seeds were set at 3 mm-radius sphere after the result from the VBM between FLE and control were obtained. Furthermore, voxel-wise, residual-based GCA evaluations were made on the mask of the grey matter using the REST toolbox (http://www.restfmri.net).

Besides, altered connections with respect to the structural changes in both FLE and healthy controls were estimated. The causal parameters; *F*_*X* → *Y*_, *F*_*Y* → *X*_ and Δ*F* representing the inflow (to seed), outflow (from seed) and out-inflow (net flow) were also computed. Here, two maps were obtained between the seed and each voxel within the grey matter mask, then the resultant inflow, outflow and the net flow were further transformed to z-score to improve normality which were used for statistical analysis. In order to find the differences between FLE and healthy control further, two sample t-test were calculated to identify the causality between the two groups (*p* < 0.05, FWE corrected), while controlling for age and gender.

### Correlation analysis between the measurements and clinical scores

For us to ascertain the altered effective relationship between statistically significant GCA areas associated with seizure in FLE, we performed partial correlation analyses between the causal areas as defined by the GCA with clinical scores (duration of epilepsy). Table 1 shows the demographic information of FLE patients.

## Results

### Voxel-based morphometric analysis

**Fig. 1:**
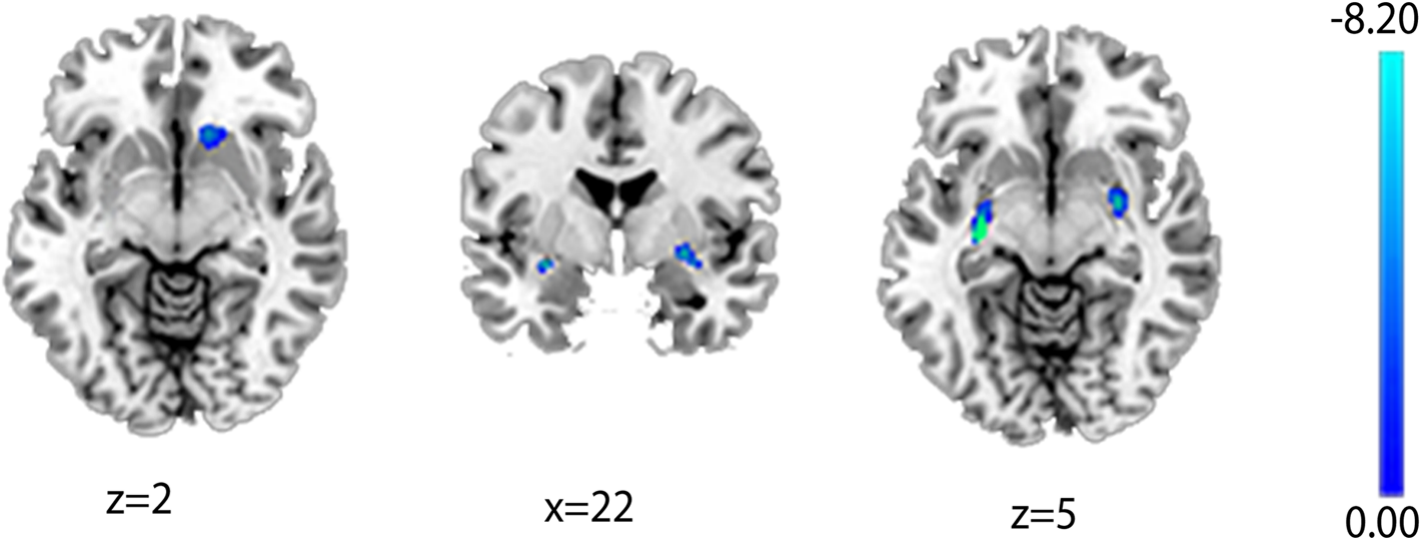
Decrease gray matter volume in FLE patients, *p* < 0.05, FWE corrected

Compared with controls, FLE patients showed significantly decrease gray matter in bilateral putamen and right caudate (*p* < 0.05, FWE corrected). The results are showed in Table 2 and Fig. 1.

**Table 2:** The differences of gray matter volume between FLE and HC

Region	L/R	MNI coordinates			Peak t	Cluster Size
		X	Y	Z		
Caudate	R	13	21	−9	−7.0762	80
Putamen	L	−30	−16	5	−8.1771	136
	R	28	−4	2	−6.7432	44

### Granger causality analysis

**Fig. 2:**
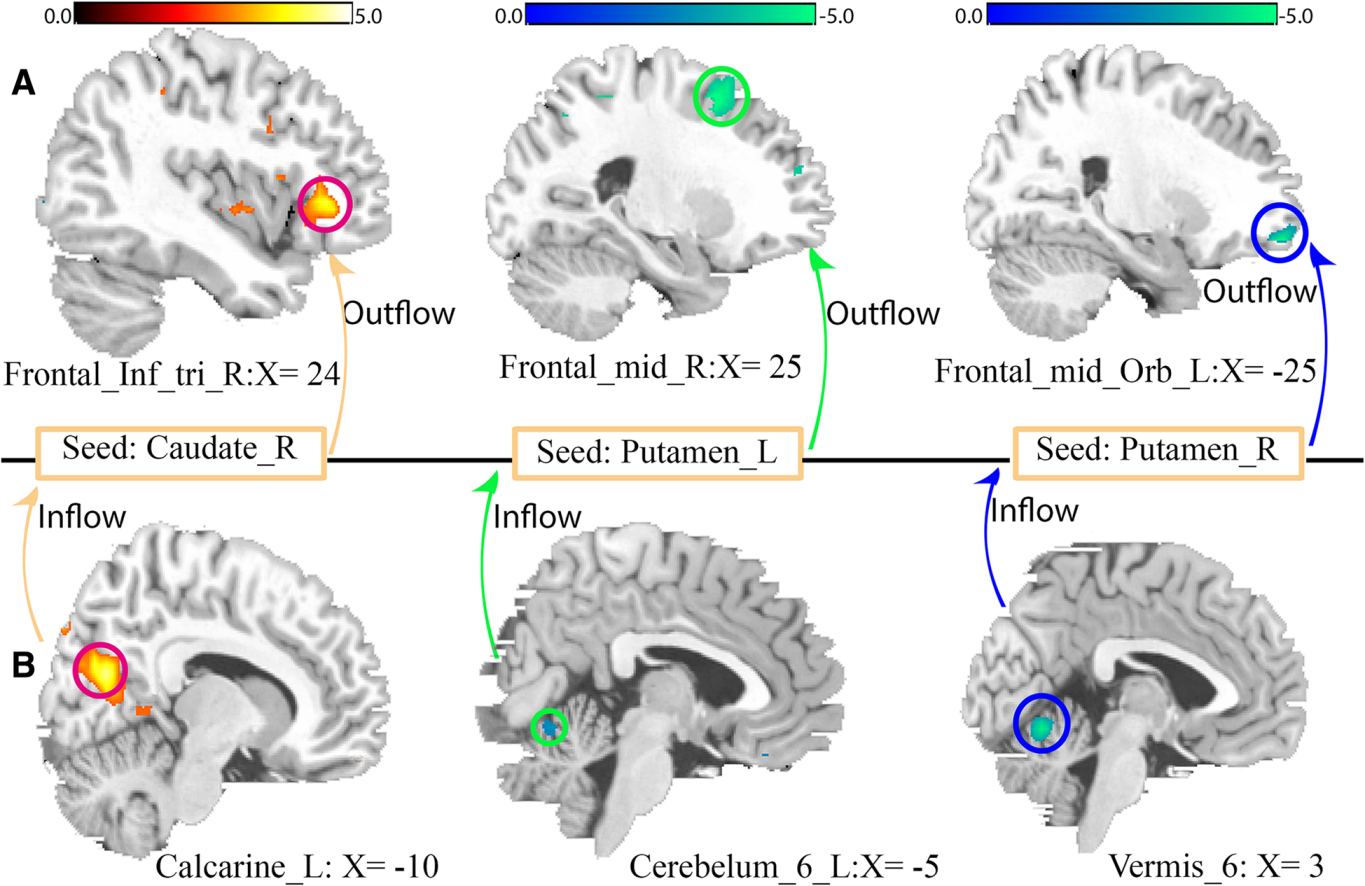
Causal connectivity differences between FLE and Healthy controls. **a** Outflow: Caudate_R (seed) to Frontal_inf_tri_L, Putamen_L (seed) to Frontal_mid_R and Putamen_R (seed) to Frontal_mid_orb_R; (**b**) Inflow: Caudate_R (seed) to Calcarine_L, Putamen_L (seed) to Cerebelum_6_L and Putamen_R (seed) to Vermis_6. *p* < 0.05, FWE corrected

Compared with controls, FLE patients revealed significant increased and decreased *F*_*x* → *y*_ (outflow) from the right caudate (seed region) to inferior frontal gyrus-triangular and from bilateral putamen (seed regions) to right middle frontal gyrus and frontal gyrus medial-orbital, respectively, as shown in Fig. 2 A. Also, significant increased and decreased *F*_*y* → *x*_ (inflow) from left calcarine to right caudate and from left cerebellum_6/vermis_6 to bilateral putamen, respectively as shown in Fig. 2 B. Table 3 shows the tmaps information of both seed and voxel including their cluster sizes.

**Table 3:** GCA based on the seed at right caudate and bilateral putamen

	Seeds	Target Areas	MNI coordinate			Peak T	Cluster Size
			x	y	z		
Outflow	Caudate_R	Frontal_inf_tri_R	24	32	3	5	270
	Putamen_L	Frontal_mid_R	25	13	52	−4	540
	Putamen_R	Frontal_mid_orb_L	−25	57	−12	−5	135
Inflow	Caudate_R	Calcarine_L	−10	−68	20	−5	810
	Putamen_L	Cerebelum_6_L	−5	− 67	−11	− 6	945
	Putamen_R	Vermis_6	3	− 66	−10	−5	1080

### Correlation analysis between GMV, effective connectivity and clinical scores

**Fig. 3:**
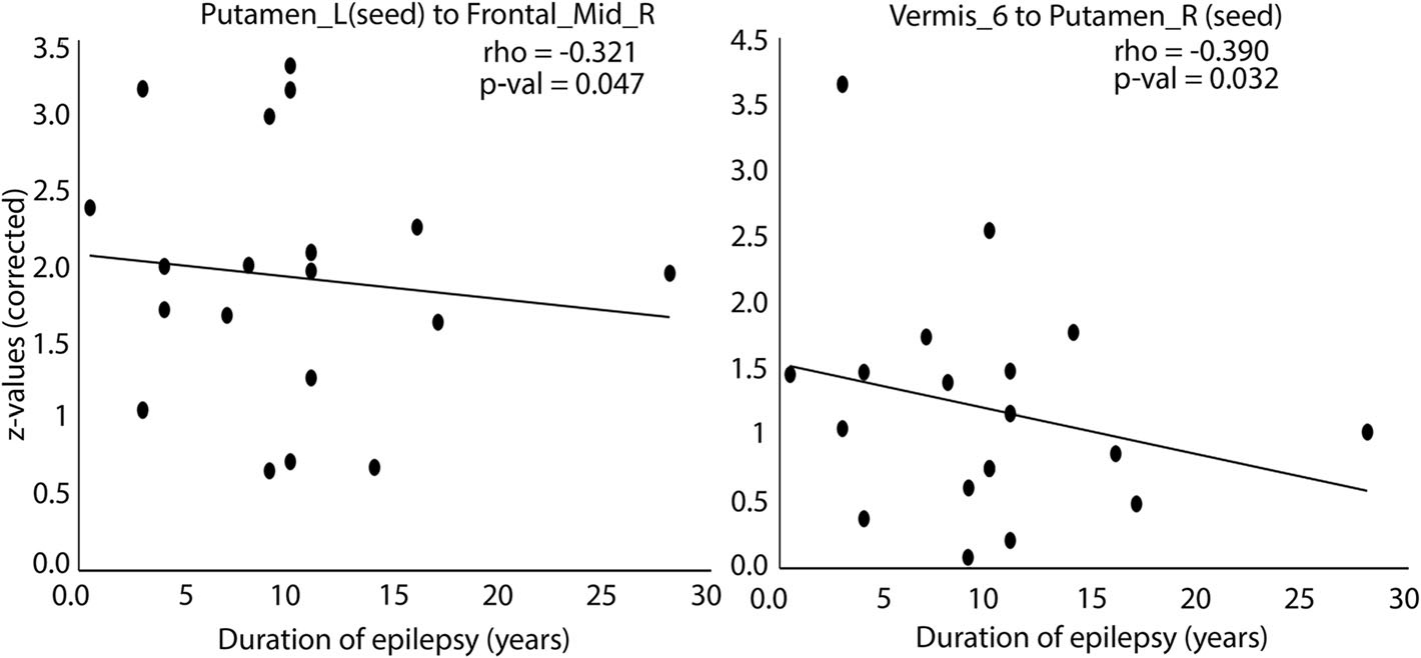
Partial correlations between GCA and duration of epilepsy. z-scores, Duration of epilepsy (years), *r*: partial correlation coefficient *p*: *p*-value

We examined the correlation between effective connectivity and the duration of epilepsy for each FLE patient. We calculated the paired correlation using partial correlation analysis while controlling for gender. Here, we found significant negative relationship between causal outflow from left putamen caudate to frontal gyrus medial-orbital (*r* =  − 0.321, *p* = 0.047). Negative causal inflow was also found from vermis to the seed region of right putamen (*r* =  − 0.390, *p* = 0.032). Moreover, we did not find any significant relation between the altered GMV and the clinical score. Figure 3 shows the correlation between GCA and clinical scores (duration of epilepsy).

## Discussion

Based on our structural and causal analysis, there were two main findings: firstly, both the caudate and the putamen exhibited reduced GMV in FLE patients. Here, compared with healthy controls, FLE patients showed significant decrease deficit in bilateral putamen as well as the right caudate. Secondly, significantly enhanced causal outflow from right caudate (seed region) to Inferior frontal gyrus-triangular and decreased outflow from bilateral putamen (seed regions) to right middle frontal gyrus and frontal gyrus medial-orbital were found. In addition, significantly enhanced causal inflow from left calcarine to right caudate (seed region), decreased inflow from left cerebellum and vermis to bilateral putamen were also found, respectively. Besides, interestingly the causal outflow from left putamen to frontal gyrus medial-orbital, and the inflow from vermis to right putamen significantly correlated with duration of epilepsy. This indicated the severity of the abnormality with regards to changes in the GMV.

### Structural deficit in FLE

In this study, the structural changes found in FLE patients were located in the caudate/putamen. These seed areas belong to the BG, which is known to be involved in variety of motor-related functions, including motor selection, preparation, and execution [29]^,^ and whose dysfunction are mostly associated with movement. In epilepsy, the BG has generally been proposed to play significant roles in the regulation and propagation of epileptic discharges [30]. The current study shows reduced GMV in the bilateral putamen and caudate of FLE patients, which shows an impairment to various motor related processes. Accordingly, the putamen works together with several other areas of the brain to provide motor abilities such as coordinating motor learning, performance of task, preparation and movement sequence [31, 32]. The evidence of these areas’ involvement in motor activity in epilepsy is consistent with our findings, which provides complimentary information about motor processes in FLE. The structures in the BG such the putamen and caudate have also been implicated in some symptom of frontal lobe seizure; these include an onset related problem, with motor symptoms including frowning, and complex automatisms (such as kicking and pelvic thrusting) and vocal manifestation (such as laughing and yelling or speech arrest) [33]. The contribution of the putamen and caudate are more identified to be major components [34], and are important in epileptic symptoms.

Furthermore, the inability of FLE patients to control voluntary movement during seizures are associated with findings in the putamen and caudate. This suggests that, the structural alteration implicates especially in controlling movement during automatisms. In addition, the caudate is known to be responsible for incorporating spatial information with motor performance preparation. Also, the caudate is suggested to monitor the integration of sensory-motor conversion, this support working memory development [35] in FLE. Therefore, our findings suggest important implications of GMV atrophies in FLE and also provide more insight in what might be an indicator to understanding FLE mechanisms.

### Altered effective connection in FLE

Here, the increased causal outflow from the caudate to the inferior frontal gyrus-triangular indicates an abnormal connectivity consistent with alterations in the higher cognitive systems, including the default mode and the motor process [36]. The caudate/putamen are important for the regulation of epileptic discharges [37]. Changes in patients, located within the frontal areas shows causality from the left putamen to frontal gyrus medial-orbital, this is responsible for mental dysfunctions in FLE patients [38]. The decreased causal inflow from the putamen and caudate to the executive regions suggested the modulation of subcortical nuclei to the frontal systems, which might be as a result of the structural changes in the region.

Furthermore, studies have found the cerebellar involvement in epileptic discharges [24]. The cerebellum is known to receive input from the cerebral cortex via the motor, premotor, parietal and occipital cortexes [39]. As far as we know there hasn’t been any report on the causality pattern within these areas in FLE patients. Remarkably, in the current study, decreased inflow observed from the left putamen to cerebellum and from vermis to right putamen and the increased inflow from the left calcarine (visual system) to the right caudate, respectively play a significant role in signals which are impaired in FLE especially during perceptual processes. These altered effective connectivity were consistent with altered functional connectivity between caudate/putamen and cerebellum [24]. Moreover, these areas are strongly responsible for motor coordination as well as signal organization and distribution. Thus, altered connectivity impedes the sensory inputs of motor-related perception and delivery in patients. The altered EC between the putamen/caudate and the cerebellar networks is thus consistent with functional evidences suggested in some studies of other types of epilepsy [7, 40]. In these studies, functional and structural connectivity have been implicated in the BG for some epileptic seizures [41, 42].

Here, the decreased in-flow from the vermis to the right putamen negatively correlated with duration, this suggest the causal influence contribution in duration of the disease, signifying that the longer duration may affect severity of the disorder along with the affected structural deficit. In addition, the outflow from the seed region to the frontal areas negatively correlated with duration of epilepsy, longer duration would result in altered connectivity between the putamen and hence reflect the severity of the of the atrophies in the GMV. Finally, together with the altered connectivity and deficits in the GMV, our results suggest the importance of cerebellar and frontal networks’ role in FLE and how causal flow has a profound influence on neurophysiological symptoms in FLE.

There are some limitations in this current study: First of all, the use of simultaneous EEG-fMRI, thus, interictal epileptiform discharges was found to affects the FC networks of focal epilepsy [43]. Also, the withdrawal of the antiepileptic drug by the FLE patients (for about 24 h) may have a profound effect on the connectivity due to frequent interictal discharges. Finally, neuropsychological evaluations in FLE patients were not conducted for correlation analysis of behavior with resting-state connectivity analysis.

## Conclusion

In conclusion, using VBM and GCA, the current study investigated the structural and causal influence between FLE patients and healthy controls. FLE patients show decreased GMV in caudate/putamen and altered effective connectivity from the seed regions of the caudate/putamen to the frontal areas as well as inflow from the cerebro-visual regions to the to the seeds. The findings implicate the found impairment within the motor-controlled system including the cerebellum and the caudate/putamen in FLE patients, this therefore suggest that the deficits in GMV at the caudate and putamen as well as their causality would enhance understanding in the prognosis of FLE.

## Abbreviations

BG: Basal Ganglia
EC: Effective connectivity
FC: Functional connectivity
FLE: Frontal lobe epilepsy
fMRI: Functional Magnetic Resonance Imaging
GCA: Granger Causality Analysis
GMV: Gray matter volume
ILAE: International League against Epilepsy
TLE: Temporal lobe epilepsy
VBM: Voxel-based Morphometry
